# Efficient On-Chip Separation and Labeling of Extracellular Vesicles from Whole Blood

**DOI:** 10.3390/bios16040220

**Published:** 2026-04-14

**Authors:** Jian Feng, Zhichen Li, Haoyang Shen, Rui Hao, Yifei Yang, Xi Chen, Xin Hong, Guoqiang Gu, Lin Zeng, Hui Yang

**Affiliations:** 1 Department of Biomedical Engineering, Southern University of Science and Technology, Shenzhen 518055, China; j.feng@siat.ac.cn (J.F.); zc.li5@siat.ac.cn (Z.L.); hy.shen@siat.ac.cn (H.S.); yf.yang3@siat.ac.cn (Y.Y.); 2 School of Biomedical Engineering, Shenzhen University of Advanced Technology, Shenzhen 518107, China; 3 Laboratory of Biomedical Microsystems and Nano Devices, Center for Bionic Sensing and Intelligence, Institute of Biomedical and Health Engineering, Shenzhen Institutes of Advanced Technology, Chinese Academy of Sciences, Shenzhen 518055, China; rui.hao@siat.ac.cn (R.H.); xi.chen@siat.ac.cn (X.C.); 4 Department of Biochemistry, SUSTech Homeostatic Medicine Institute, School of Medicine, Southern University of Science and Technology, Shenzhen 518055, China; hongx@sustech.edu.cn

**Keywords:** extracellular vesicles, negative magnetophoresis, high-throughput separation, tesla micromixer, on-chip labeling

## Abstract

The development of high-throughput technologies for the separation and labeling of extracellular vesicles (EVs) from whole blood is critical for downstream EV detection and analysis. However, conventional EV separation and labeling workflows are typically labor-intensive and inefficient, requiring multiple sequential processing steps. Here, we present a microfluidic platform that integrates negative magnetophoresis-based separation with mixing-enhanced on-chip labeling. The chip adopts a vertical flow channel architecture in combination with a Halbach-array magnetic field configuration, thereby overcoming the throughput limitations inherent to traditional horizontal microchannels. Parallel channels can be freely arranged above on the magnetic array to achieve ultra-high throughput processing, achieving a cell removal efficiency of 99.97% at a blood-to-sheath flow ratio of 1:5. Furthermore, by incorporating a narrow-wide channel design synergized with a herringbone–Tesla micromixer structure, the platform achieves a labeling efficiency of 91.8% within 2 min, approaching the performance of conventional 20 min incubation. This system offers both high-throughput and integration capabilities, providing a powerful technical platform for EV-related life science research.

## 1. Introduction

Cancer has remained a major threat to global health for decades. Extracellular vesicles (EVs), membranous nano particles present in the peripheral blood of cancer patients, serve as critical mediators of intercellular communication, and extensive evidence has demonstrated their involvement in cancer initiation, progression, and metastasis [1,2,3,4,5]. Consequently, EV analysis holds substantial promise for various of clinical applications, including cancer diagnosis, prognosis, and therapeutic response monitoring [6,7,8,9,10]. Achieving these objectives requires the efficient isolation of EVs from patient blood samples, followed by robust fluorescent labeling, which together constitute essential preparatory steps for downstream tracking and functional analyses.

However, the nanoscale dimensions of EVs makes their separation and purification technically challenging. Currently, differential centrifugation remains the most commonly used method for EV separation; however, it relies on ultracentrifugation, which is time-consuming, costly, and labor-intensive [6,11,12]. With the advent of microfluidic technologies, increasing efforts have focused on developing integrated and portable blood separation and analysis platforms. Owing to their miniaturized architecture and precise fluid control, microfluidic systems have demonstrated superior performance compared with conventional separation and purification methods [13,14,15,16].

In microfluidic systems, separation techniques based on physical properties—such as deterministic lateral displacement and filtration—have garnered considerable attention due to their simplicity and low cost [17,18]. However, current microfluidic strategies frequently encounter bottlenecks in throughput capacity and prone-to-clogging issues when handling complex biological matrices. For instance, Wunsch et al. reported a nano-DLD array for EV sorting at an extremely low flow rate of approximately 0.2 μL/h by controlling the gap size of nanorods [19], while Smith et al. achieved a maximum flow rate of only 900 μL/h by integrating 1024 nano-DLD arrays into a single device [20]. In recent years, negative magnetophoresis has emerged as a promising label-free and non-contact separation strategy [21,22,23,24]. By applying a magnetic field with a defined spatial distribution, non-magnetic particles experience a repulsive force perpendicular to the flow direction, enabling precise size-dependent separation. Previous studies have shown that this approach not only avoids channel blockage but also preserves the biological activity of plasma components, providing high-quality samples for subsequent analysis [25]. However, most existing negative magnetophoretic devices employ horizontal channel layouts that confine particle separation to lateral movement within a two-dimensional plane, resulting in limited throughput [26]. For instance, Leidong Mao’s group reported a label-free cell separation method using negative magnetophoresis, achieving a throughput of 1.2 mL/h. However, their design, featuring side-positioned magnets, inherently restricted parallelization with additional channels, thereby fixing 1.2 mL/h as the maximum throughput [22]. Moreover, the labeling process in that system was performed off-chip, lacking on-chip integration. In contrast, Joo H. Kang’s team achieved high-throughput EV separation via negative magnetophoresis by employing parallel channels. However, the absence of flow focusing led to EV loss, rendering its recovery rate merely comparable to that of ultracentrifugation [25].

At present, research based on microfluidic technology is increasingly tending towards the integration of different functions [27,28,29], while the seamless integration of high-efficiency separation and labeling within a compact microfluidic platform—enabling a continuous and automated separation-labeling workflow—remains a significant challenge [30]. To overcome these limitations, we present an integrated microfluidic strategy that achieves efficient blood cell removal and simultaneous rapid EV labeling directly from whole blood. The platform combines two functional modules. First, a vertically oriented flow channel, combined with a Halbach array composed of alternating long and short N52 permanent magnets, generates a strong vertical magnetic field gradient that facilitates label-free separation of blood cells and EVs. The platform ultimately achieved a throughput of 80 μL/min, with further scalability enabled by the magnet array’s overhead channel configuration. Compared to ultracentrifugation, our method eliminates the need for large-scale instruments while achieving comparable throughput. Meanwhile, the sheath-to-sample flow ratio of 5:1 maximizes the retention of EVs in the bottom region of the channel, resulting in an EV recovery rate of 69.84%. Following separation, the separated EVs are subsequently guided into a herringbone–Tesla micromixer module for rapid fluorescent labeling.

## 2. Materials and Methods

### 2.1. Chip Design

**Figure 1:**
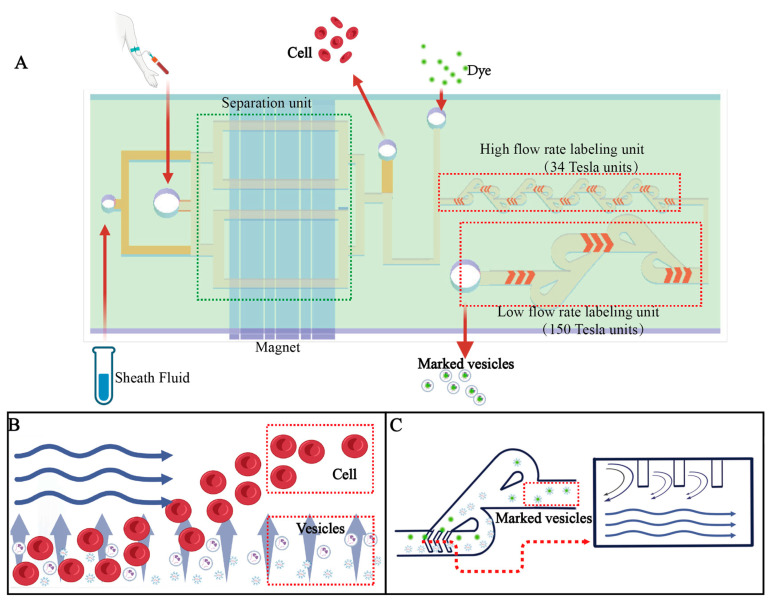
(**A**) Schematic of the microfluidic chip. (**B**) Schematic illustration of the cell–EV separation module. (**C**) Schematic illustration of the EV labeling module.

We used an integrated microfluidic platform comprising two main functional modules (Figure 1): an EV separation module and an EV fluorescence labeling module.

The separation channel consists of two inlets, with the blood sample introduced through the lower channel layer and the sheath flow through the upper layer. As the fluid pass the negative magnetophoretic separation region generated by a Halbach array, blood cells are selectively deflected toward the upper channel layer and subsequently removed through the corresponding outlet. Because the channel is positioned above the magnet array, the design readily accommodates parallel channel integration, enabling scalable throughput enhancement. The EVs, which remain in the bottom layer of the channel, are directed together with the fluorescent dye (EV Membrane Red Stains, Nano FCM, Xiamen, China) into the downstream labeling module. The labeling module consists of two sequential sections: a high-shear region composed of 34 small herringbone–Tesla mixing units to enhance membrane permeability, followed by a second region comprising 150 large herringbone–Tesla mixing units, which provide sufficient residence time for complete EV labeling.

### 2.2. Fabrication of the Microfluidic Chip

The microfluidic system was fabricated in four steps. First, the upper microchannel layer was produced by standard soft lithography, with channel heights of 180 μm for the separation module and 60 μm/180 μm for the labeling module. Second, the lower microchannel layer was fabricated using the same method, with a uniform height of 150 μm for all microchannels. Third, polydimethylsiloxane (PDMS) was cast onto a silicon wafer. For the lower microchannel layer, the PMDS was spin-coated onto the silicon wafer at 500 rpm for 20 s and subsequently cured at 80 °C for 20 min to form a 180 µm PDMS film, so as to control the distance between the magnet and the channel to be 180 µm. The magnet array was then positioned on the EV separation module, and a subsequent layer of PDMS was cast to encapsulate the magnets. Finally, the upper and lower microchannel layers were aligned and bonded together by plasma bonding after mold release.

### 2.3. Experimental Setup

To evaluate the performance of the separation system, a simulated whole blood sample containing tumor cell-derived EVs was prepared. The sample comprised commercially obtained rabbit whole blood (Zhengzhou Pingrui Biotechnology Co., Ltd., Zhengzhou, China), tumor-derived EVs and a 2% dilution of ferrofluid (Ferraheme, a US Food and Drug Administration-approved intravenous iron preparation from AMAG Pharmaceuticals, Waltham, MA, USA). The rabbit whole blood was pre-processed to remove endogenous EVs to preclude interference with subsequent analysis. Since the separation throughput is adjustable, the labeling efficiency of the labeling module at different flow rates was first investigated using nanoflow cytometry (NanoFCM, Xiamen, China). Subsequently, under the optimized flow rate, the effect of the sheath-to-blood flow rate ratio in the separation module on cell removal efficiency was evaluated by flow cytometry (FCM, CytoFLEX S, Beckman Coulter, Atlanta, GA, USA).

### 2.4. Sample Collection and Preparation

Human glioma U251 cells (National Cell Line Resource Platform, Beijing, China) were cultured in Dulbecco’s modified Eagle medium (DMEM; Thermo Fisher Scientific, Waltham, MA, USA) supplemented with 10% fetal bovine serum and 100 U·mL^−1^ penicillin–streptomycin at 37 °C in a humidified incubator with 5% CO_2_.

For the experiments validating EV separation and labeling, EVs were isolated from cell culture supernatants via ultracentrifugation. The cell suspension was first centrifuged at 3000× *g* for 30 min at 4 °C to remove cell and cell debris. The resulting supernatant was carefully transferred using a Pasteur pipette to an Ultra-Clear tube (26.3 mL, Beckman Coulter, Brea, CA, USA) and subjected to ultracentrifugation at 100,000× *g* for 70 min at 4 °C. After discarding the supernatant, the pellet was resuspended in PBS and centrifuged again at 100,000× *g* for 70 min at 4 °C to wash the vesicles. Finally, the purified EV pellet was resuspended in PBS for subsequent experiments. For blood-spiking experiments, EVs were added to rabbit blood at a final concentration of 1 × 10^9^ particles/mL. For fluorescence labeling, 100 μL of diluted dye (1:1000) was added to 100 μL of EV suspension (1 × 10^9^ particles/mL). After thorough mixing, the mixture was incubated at 37 °C for 20 min in the dark. For on-chip staining, the dye was likewise diluted 1000 times prior to use. The size distribution and concentration of EVs were characterized using nano-flow cytometry (NanoFCM, Xiamen, China). Prior to measurement, the instrument was calibrated with silica nanospheres of known diameters.

### 2.5. TEM Characterization of EVs

The morphologies of the EVs were measured by transmission electron microscopy (TEM, FEI Tecnai Spirit T12, Thermo Fisher Scientific, Waltham, MA, USA). In brief, 100 μL of EV samples were firstly fixed with 25 μL of 4% paraformaldehyde (*v*/*v*); then, 10 μL samples were separately pipetted onto the grids and incubated for 20 min for absorption at room temperature (RT), followed by washing the sample with filtered water (0.1 μm) once; finally, the samples were negatively stained with 2% aqueous uranyl acetate for 10 min and dried out at RT overnight. All the EV samples were prepared following the above processes and analyzed using TEM.

## 3. Results

We first present a theoretical analysis of the forces acting on particles within the separation microchannel and the mixing principles of the herringbone–Tesla micromixer, followed by experimental validation of the system performance.

### 3.1. Theory of Separation

As whole blood enters the microchannel, sheath-flow-induced hydrodynamic focusing confines it to a narrow stream. Within this stream, particles are transported by hydrodynamic drag $F_{d}$ [31]. Upon entering the separation region, the particles additionally experience a negative magnetophoretic force $F_{m}$ [32]:

(1) $$F_{m}=\frac{V_{p}\left(\chi_{p}-\chi_{f}\right)}{\mu_{0}}\left(B\cdot \nabla \right)B$$

where $V_{p}$ is the volume of the particle, $\mu_{0}$ is the permeability of free space, B is the magnetic induction, and $\chi_{p}$ and $\chi_{f}$ are the magnetic susceptibilities of particle and ferrofluid, respectively. The negative magnetophoretic force $F_{m}$ is proportional to the particle volume.

The particles of different sizes eventually enter different outlets following different trajectories that are determined by these two forces. The motion control equation is

(2) $$m_{P}\frac{d\mu_{p}}{dt}=F_{m}+F_{d}$$

Based on Equations (1) and (2), the particle trajectories can be calculated by the simulation software.

### 3.2. Separation Simulation

**Figure 2:**
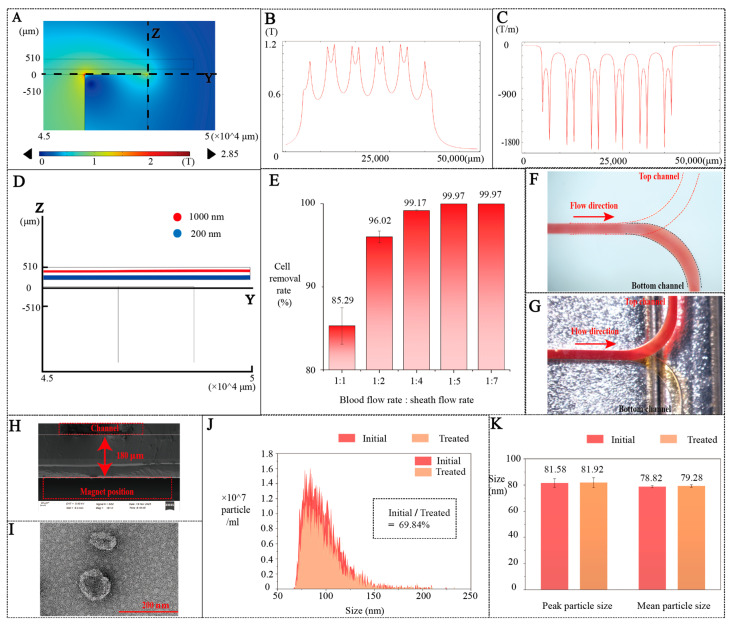
(**A**) Magnetic field distribution. (**B**) Magnetic flux density along *y*-axis at z = 180 μm. (**C**) Magnetic flux density gradient along *y*-axis at z = 180 μm. (**D**) FEM simulation of cell separation (1000 nm and 200 nm particles). (**E**) The cell removal rate under different flow rate ratios of blood and sheath fluid. (**F**) Cell outflow from the lower channel (blood-to-sheath flow ratio of 1:5, total flow rate: 80 μL/min). (**G**) Cell outflow from the upper channel under magnetic field. (**H**) Distance from the magnet to the channel (SEM image, the asterisk in sub-figure (**H**) denotes a calibrated scale bar, verified with a standard sample to ensure dimensional accuracy.) (**I**) TEM image of EVs collected from outlet. (**J**) Size distribution profiles of the initial sample and the sample collected after separation. The recovery rate of particles after separation was 69.84%. (**K**) Comparison of peak size and mean size between the initial sample and the sample collected after separation. Peak size remained comparable (81.58 nm vs. 81.92 nm), while mean size remained comparable (78.82 nm vs. 79.28 nm).

A numerical model of the separation region was established in COMSOL(6.2) Multiphysics to analyze the magnetic field distribution (Figure 2A). The magnetic field and its gradient along the *y*-axis (at z =180 μm, x = 0 μm) are shown in Figure 2B and Figure 2C, respectively. The simulation results indicate that the Halbach magnet array generates a magnetic field exceeding 1.24 Tesla within the separation channel, with a maximum magnetic field gradient of 1920 T/m.

Building on the magnetic field simulation, we further simulated the trajectories of 200 nm and 1000 nm particles in the separation region, as shown in Figure 2D. The 1000 nm particles experience a significant negative magnetophoretic force due to their larger size, which drives them toward the top of the channel. In contrast, the 200 nm particles are subjected to a negligible force and thus remain confined near the channel bottom.

### 3.3. Separation Experiments

In accordance with our previous research, a ferrofluid concentration of 2% was confirmed to have no detrimental effect on plasma quality [26]. In this study, the sheath-to-blood flow rate ratio was first optimized to achieve two objectives: (1) confining the blood stream to the lower layer of the channel (Figure 2F) to maximize EV recovery (Figure 2G); (2) maximizing the removal efficiency of cells larger than 1 µm.

To quantify the particles collected from the two outlets, here we closed the fluorescent dye inlet, and the EVs collected at the outlet of the labeling module were only used to verify the separation efficiency. Particle counts at each outlet were quantified by flow cytometry under different sheath-to-blood flow rate ratios. The results are summarized in Figure 2E. When the magnet is 180 µm away from the bottom of channel (Figure 2H), a flow rate ratio of 1:5 was found to not only achieve effective flow focusing but also realize a cell removal rate of 99.97%. Both theoretical and experimental results conclusively demonstrate the high separation performance of the system.

In addition, the recovery rate of EVs reached 69.84% with well-preserved structure integrity (Figure 2I,J). Importantly, no significant changes in particle size distribution were observed before and after separation. As shown in Figure 2J, the concentration profiles across different particle sizes remained largely unchanged between the initial sample and the post-chip sample. Moreover, Figure 2K further confirms that both the peak size and mean size exhibited minimal variation, indicating that the separation process did not alter the intrinsic size characteristics of the EVs.

### 3.4. Mixing Theory

**Figure 3:**
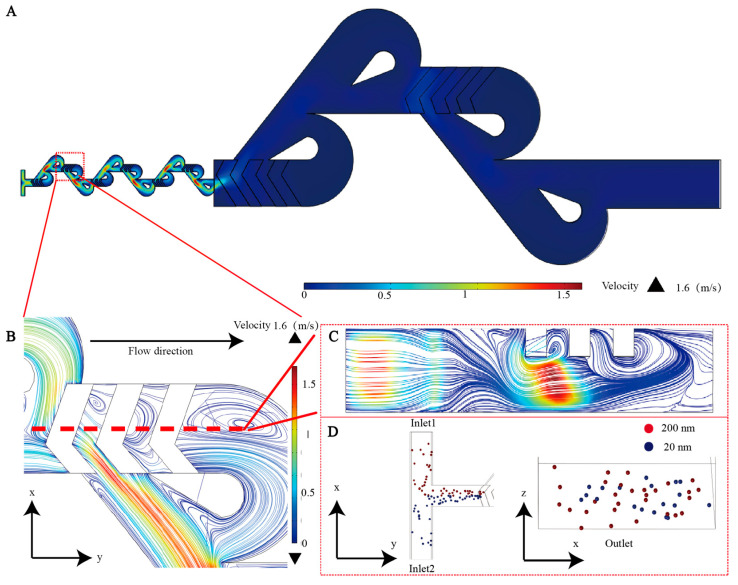
(**A**) FEM simulation results at a flow rate of 80 μL/min. (**B**) Horizontal streamline of the sunken herringbone structure. (In fluid dynamics simulations, dense streamlines indicate regions of high flow velocity, whereas closed or swirling streamlines suggest the presence of vortices.) (**C**) Streamline diagrams of the cut surface of the sunken herringbone. (**D**) Simulated particle distribution at the inlet and outlet using 20 nm and 200 nm particles in the particle tracing module to demonstrate mixing efficiency.

The Tesla micromixer operates based on the split-and-recombine (SAR) principle. The main channel divides into two asymmetric sub-channels. Due to the Coanda effect, the fluid flows along the curved surfaces [33]. The separated streams traverse curved and straight subchannels, respectively, before recombining. The collision of the two streams generates chaotic advection, which enhances the mixing efficiency [34,35,36]. To further improve mixing performance, herringbone structures from our previous micromixer design were integrated into the conventional Tesla mixer [37]. These structures promote lateral flow and vertical vortex formation and thereby significantly enhancing mixing (Figure 3B–D).

Based on the simulation results, we implemented a hybrid configuration comprising scaled-down and scaled-up herringbone–Tesla micromixers connected in series to achieve efficient labeling. The EVs first pass through the scaled-down mixers (main channel: 80 µm wide × 60 µm high), where increased shear enhances membrane permeability and promotes incorporation of lipophilic dyes. At a flow rate of 80 µL/min, the maximum internal flow velocity reaches 1.6 m/s (Figure 3A). The flow then enters the scaled-up mixers (main channel: 500 µm wide × 180 µm high), where the reduced velocity prolongs the interaction time between the EVs and dyes, further improving the labeling efficiency.

### 3.5. Labeling Experiments

**Figure 4:**
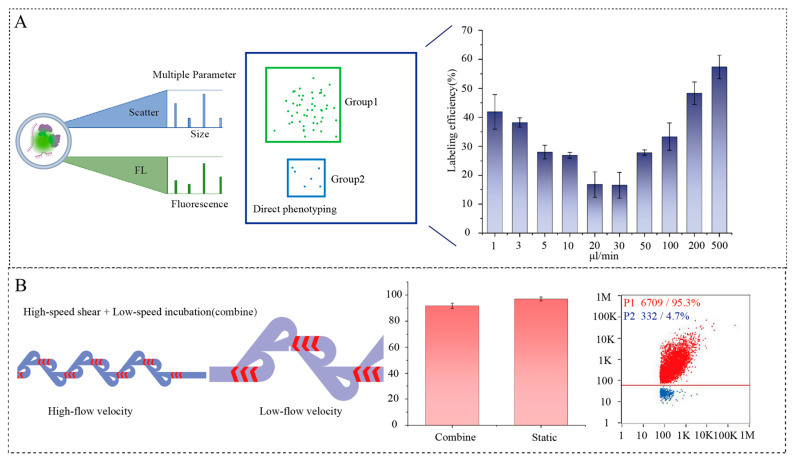
(**A**) Labeling efficiency of EVs at different flow rates (Original Chip: main channel: 200 µm wide × 150 µm high, testing by nanoparticle flow cytometry). (**B**) Comparison of labeling efficiency of the chip with narrow-wide channels versus that of normal temperature incubation staining.

We first investigated the relationship between labeling efficiency and flow rate using our previously developed mixing chip (main channel: 200 µm wide × 150 µm high) (Figure 4A) [37]. The results indicated that at flow rates below 30 µL/min in the main channel, labeling efficiency decreased with increasing flow rate due to insufficient EV–dye interaction time. Conversely, at flow rates above 30 µL/min, increased shear stress enhanced membrane permeability and accelerated dye incorporation, thereby improving labeling efficiency. However, under both low and high flow rate conditions, the labeling efficiency remained below 60%, substantially lower than that achieved by conventional incubation. These results suggest that both shear force and residence time are critical for optimizing labeling efficiency—neither increasing shear force alone nor extending residence time alone is sufficient.

To overcome these limitations, we designed a hybrid herringbone–Tesla micromixer with narrow-wide herringbone–Tesla micromixers connected in series for on-chip EV labeling (Figure 4B). In this design, EVs are first subjected to high shear stress in the narrow section (main channel: 80 µm wide × 60 µm high), which increases membrane permeability. They then flow into a wider channel section (main channel: 500 µm wide × 180 µm high), where the reduced flow velocity provides sufficient residence time for dye-membrane interaction. Finally, following the introduction of the spiked sample at the inlet, the EVs collected from the outlet were analyzed via nanoparticle flow cytometry, which demonstrated that this combined strategy achieved a labeling efficiency of 91.8% within just 2 min, comparable to that of a conventional 20 min off-chip incubation, while maintaining a high.

## 4. Discussion

In this study, we developed and validated an integrated microfluidic platform that combines negative magnetophoresis-based separation with mixing-enhanced labeling, achieving the high-throughput and high-efficiency isolation of EVs from whole blood together with rapid fluorescent labeling. Under an optimized blood-to-sheath flow rate ratio of 1:5, the platform achieved a 99.97% removal efficiency for cells larger than 1 µm and a 69.84% recovery rate for EVs, while also providing a scalable framework for further enhancing system throughput. For labeling, the platform achieved a 91.8% labeling efficiency within 2 min, comparable to conventional 20 min off-chip incubation, while maintaining a high processing throughput of 80 µL/min.

Compared with existing methods, our platform demonstrates significant advantages. Owing to the bottom-positioned magnet array beneath the microchannel, parallel channels can be freely arranged in the horizontal direction, offering the potential for further throughput enhancement. Furthermore, by focusing the sample near the bottom of the channel, a higher EV recovery rate was achieved. More importantly, most microfluidic studies merely focus on the optimizing a single function, namely either separation performance enhancement or labeling process improvement; by contrast, our platform achieves the seamless integration of both separation and labeling in a continuous workflow, eliminating sample loss associated with transfer and significantly reducing processing time. Despite the abovementioned merits, this proposed platform still possesses certain limitations. The labeling module employs a lipophilic dye suitable for universal membrane staining, whereas specific labeling such as antibody-mediated is required for applications demanding specific phenotyping. In addition, given that the separation principle of this method relies on particle size difference, and lipoproteins share overlapping size and density ranges with EVs, the co-isolation of lipoproteins may occur in the final collected sample, making the purity of EVs dependent on the initial sample composition. To tackle these challenges, future research efforts can be devoted to incorporating additional functional modules such as immunoaffinity capture units (e.g., immobilization of tetraspanin antibodies) to further elevate the purity of isolated EVs. Overall, this highly integrated and high-throughput microfluidic system offers a robust technological platform for EV-related life science research.

## Data Availability

The data presented in this study are available on request from the corresponding author. The data are not publicly available due to privacy and ongoing research restrictions.
